# Reversed Phase SPE and GC-MS Study of Polycyclic Aromatic Hydrocarbons in Water Samples from the River Buriganga, Bangladesh

**DOI:** 10.1155/2014/234092

**Published:** 2014-10-29

**Authors:** Md. Saddam Nawaz, Farhana Khanam Ferdousi, Mohammad Arifur Rahman, A. M. Shafiqul Alam

**Affiliations:** ^1^Department of Chemistry, University of Dhaka, Dhaka 1000, Bangladesh; ^2^Quality Assurance Department, ACI Ltd., Narayanganj 1400, Bangladesh

## Abstract

Polycyclic aromatic hydrocarbons (PAHs) are semivolatile organic compounds (SVOCs) categorized as persistent organic pollutants (POPs). PAHs are ubiquitous in terrestrial, atmospheric, and particularly aquatic environments throughout the world and have been detected in lakes, ground waters, and rivers. This research work involved the analysis of five PAHs, anthracene, fluorene, naphthalene, phenanthrene, and pyrene, in water sample collected from the river Buriganga, Bangladesh. The extraction of water samples was carried out by reversed phase solid-phase extraction (RP-SPE) technique with C-18 SPE cartridges. A solvent mixture of dichloromethane and hexane (1 : 2) with a flow rate of 0.5 mL/min was used as eluent. Percentage recoveries of five PAHs for this technique were in the range of 81.47 ± 1.16 to 98.60 ± 0.61%. PAHs quantification was achieved by using an ion trap gas chromatography mass spectrometer (GC-MS) interfaced to gas chromatography (GC) equipped with a fused silica capillary column. Helium was used as carrier gas with a flow rate of 1.0 mL/min. The commonly detected PAH compounds in the river water were anthracene, naphthalene, and phenanthrene at the concentration ranges of 0.451 to 3.201, 0.033 to 3.1131, and 0.320 to 2.546 *μ*g/mL, respectively. The results reflect that PAHs presented in this river water were mostly from petrogenic and pyrogenic sources.

## 1. Introduction

Polycyclic aromatic hydrocarbons (PAHs) are one of the most important classes of environmental pollutants that contain two or more fused aromatic (benzene) rings [1]. PAHs are semivolatile organic compounds (SVOCs) and are also regarded as persistent organic pollutants (POPs) in the environment due to their inherent hydrophobicity. Their presence in surface water or groundwater is an indication of a source of pollution. Generally, PAHs may be divided into two groups based on their molecular structure: low-molecular-weight (LMW) PAHs having three or fewer aromatic rings, which have significant acute toxicity to aquatic organisms, and high-molecular-weight (HMW) PAHs having four or more aromatic rings, which are not acutely toxic to aquatic organisms but several of them are carcinogenic. Solubility of PAHs decreases with increasing ring number (molecular weight), but persistency, and settles out with organic and inorganic particles in the aquatic environment increases with increasing ring number and degree of condensation [2, 3]. PAHs are only slowly biodegradable under aerobic conditions and are stable to hydrolysis [4].

PAHs occur in the environment both naturally and anthropogenically. Formation of PAHs in nature includes high temperature pyrolysis of organic materials, low to moderate temperature diagenesis of sedimentary organic material to form fossil fuel, and direct biosynthesis by microbes and plants [5]. Forest fires and volcanic activity are also natural sources of PAHs, but their contribution is less significant to the overall PAH emission [6].

Pyrogenic and petrogenic sources are two major origins of anthropogenic PAHs in the environment. Pyrogenic PAHs are formed as trace contaminants by the incomplete combustion of organic materials during industrial and other human activities, combustion of natural gas, vehicle traffic, treated wood, cooking, tobacco smoking, and untreated industrial waste. One of the major anthropogenic sources of PAHs in urban runoffs is deterioration of asphalt pavement surfaces and car tires, which leads to passage of the compounds to runoff waters [7]. Crude and refined petroleum releases are a petrogenic source of PAHs.

Aquatic contamination of PAHs generally includes oil spillage and leakage of PAH-containing fluids (e.g., waste oils, gasoline, etc.), domestic sewage, stormwater runoff, urban runoff, discharges originating from landfills, and use of creosoted pilings for dockyards and other shoreline structures.

The importance of PAHs in the environment is discussed because of their persistent, toxic, mutagenic, and carcinogenic characteristics [8]. Some PAHs have been classified as priority pollutants [9]; sixteen of the common or so “unsubstituted” PAHs, naphthalene, acenaphthylene, acenaphthene, fluorene, phenanthrene, anthracene, fluoranthene, pyrene, benzo[a]anthracene, chrysene, benzo[b]fluoranthene, benzo[k]fluoranthene, benzo[a]pyrene, dibenz[a,h]anthracene, benzo[g,h,i]perylene, and indeno[1,2,3-cd]pyrene, typically analyzed in standard contract laboratory scans have been listed by the United States Environmental Protection Agency (US EPA). Scientific, regulatory, and public interest concern about PAHs initially focused on their ability to cause cancer, but more recently concern has turned to their interference with hormone systems and their potential effects on reproduction, as well as their ability to depress immune function.

The most common techniques used to determine PAHs are gas chromatography (GC), coupled with mass detector [10–12], and high performance liquid chromatography (HPLC) [13–15]. According to the official method of the Environmental Protection Agency (EPA), PAHs are determined in drinking water by HPLC coupled with ultraviolet (UV) or fluorescence detector [16].

Because of low concentration levels to be quantified in water samples, an enrichment and cleanup procedure is needed before chromatographic analysis. For PAHs, the most acceptable preconcentration technique is solid-phase extraction (SPE). There has been reported different application of SPE on the preconcentration of PAHs from water [17–19] precipitation [20] and aerosol [21].

SPE is a sample preparation technique that is becoming increasingly popular, because unlike liquid-liquid extraction (LLE) it is easy to perform, is rapid, is automated, and does not require large quantities of toxic organic solvents and analysis time can be decreased significantly. Another advantage of SPE over LLE is complete, phase separation and good quantitative recoveries.

The river Buriganga is the main river flowing through Dhaka city, capital of Bangladesh, and is economically very important to the country. Today, the river Buriganga is afflicted by the various problem of pollution. Apart from untreated industrial and household wastes, burnt motor oil, lubricant, and dyeing chemicals that are possible sources of PAHs are also contributing greatly to the pollution in this river.

**Figure 1:**
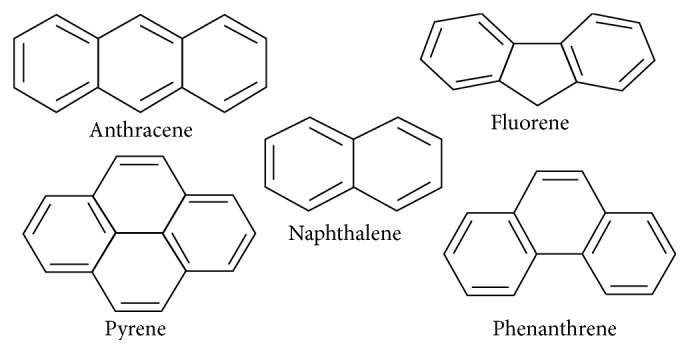
Chemical structures of five targeted PAHs.

Our research study involved determining the concentration level of five PAHs, anthracene (ANT), fluorene (FLU), naphthalene (NAP), phenanthrene (PHE), and pyrene (PYE), from the river Buriganga, as shown in Figure 1. In this monitoring work, a simple, suitable, and rapid reversed phase extraction procedure by use of solid-phase extraction (SPE) processor with C-18 sorbent was illustrated for the extraction of target PAHs from the river water sample. After extraction through SPE processor, identification and determination were carried out by a published validated GC-MS method, which was applied to determine PAHs extract from edible oil as discussed by Hossain and Salehuddin [22]. In our work, we have applied the identical chromatographic procedure to determine our extracted PAHs from river water samples.

## 2. Material and Methods

### 2.1. Chemicals and Reagents

ANT, FLU, NAP, PHE, and PYR standards were purchased with the purity of 98.0 to 99.0% (Sigma-Aldrich). Analytical or HPLC grade solvent hexane (Scharlau), acetone (Scharlau), dichloromethane (Scharlau), and methanol (Scharlau) were used to carry out all the experiments. Purified deionized water (D.I. water) which does not contain any measurable quantities of any target analytes or interfering compounds was used.

Sample preservation reagents L-ascorbic acid (Sigma-Aldrich), ethylenediaminetetraacetic acid trisodium salt (Sigma-Aldrich), diazolidinyl urea (Sigma-Aldrich), and premixed crystals of tris(hydroxymethyl)aminomethane (Merck) and tris(hydroxymethyl)aminomethane hydrochloride (Merck) were all analytical reagent grades. Pure anhydrous sodium sulfate (Merck) was freed from interfering organic substances and moisture by heating at 400°C for 4 hours in a muffle furnace.

All required glass apparatuses were cleaned with water using detergent, rinsed about six times with water and then twice with D.I. water, and finally dried in oven. Polytetrafluoroethylene (PTFE) lined screw caps of vials were cleaned by ethanol and heated at 105°C for 4 hours before use.

### 2.2. Instruments and Operating Conditions

A Varian standard CEREX SPE processor was used for extraction of target PAHs from the river water samples. The 10 mL cartridges, packed with Bond Elut C-18 (500 mg) of Varian, were used and the extraction was done by positive pressure by N_2_ gas. The pressure controller was turned on in the upper and lower positions to monitor and preadjust the desired pressure in each position. The lower regulator was in the range of 0 to 30 psi and was used for drying the cartridges. The upper regulator was in the range of 0 to 10 psi with greater sensitivity of adjustment. Both knobs were used to increase the pressure when turned clockwise and to decrease pressure when turned counterclockwise.

The GC-MS analysis was carried out using total ion monitoring mode on a Varian Saturn ion trap 2200 GC-MS mass spectrometer interfaced to a Varian CP 3800 (USA) gas chromatograph. The VF-5 capillary column (30 m length, 0.25 mm I.D., and 0.25 *μ*m film thickness; Varian) was used. Helium was used as carrier gas with a flow rate of 1.0 mL/min. The temperatures of injector and mass transfer line were set at 275°C and 300°C, respectively. Ions were obtained by electron ionization (70 eV) mode. Injected volume was 0.2 *μ*L in the split less mode. The oven temperature was programmed to rise from 50 to 200°C at 8°C/min, then held isothermal for 20 min., and finally raised to 300°C at 10°C/min. Identification of compounds was based on GC retention time on VF-5 capillary column, computer matching of mass spectra with standards.

### 2.3. Preparation of Standard

Different concentrations of ANT, FLU, NAP, PHE, and PYE (0.50 to 6.50 *μ*g/mL) were prepared from the stock solutions by dilution of this standard mixture into n-hexane to establish the calibration curves. To avoid volatilization and photodegradation, standard mixture was stored at 4 ± 1°C in darkness.

### 2.4. Methodology

The quantitative determination of PAHs was done by the external calibration curve method. Each PAH was separately quantified using five different concentrations (0.50 *μ*g/mL, 1.25 *μ*g/mL, 2.50 *μ*g/mL, 5.00 *μ*g/mL, and 6.5 *μ*g/mL) of mixed standard solution. Data acquisition was carried out at selected ion monitoring (SIM) mode. The linearity was evaluated by peak area versus concentration, which was calculated by linear regression analysis.

### 2.5. River Water Samples

**Figure 2:**
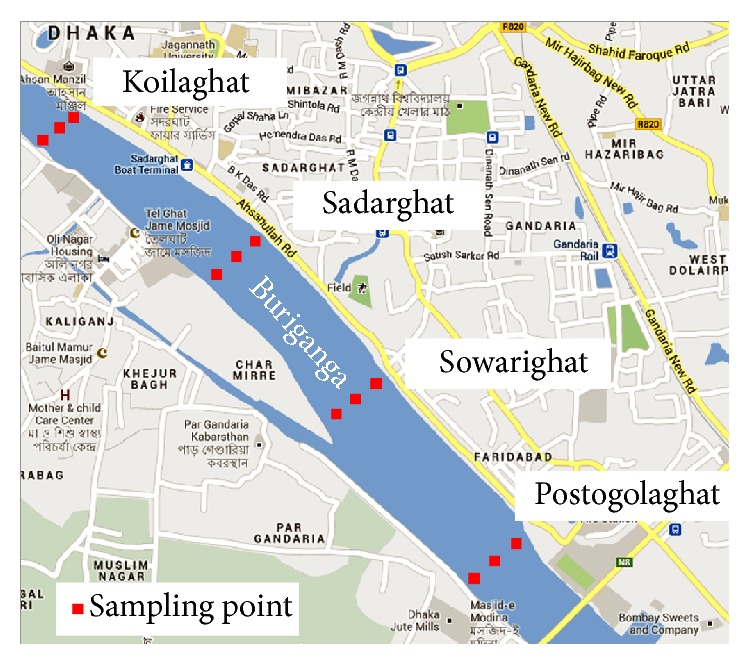
Map of the Buriganga river showing sampling stations and location of collection points of water samples at 20 cm depth.

About 12 liters of water sample was collected on March 25, 2010, and once more on July 7, 2011, in 12 amber glass bottles from the Buriganga river, from four different stations, Postogolaghat, Sadarghat, Sowarighat, and Koilaghat, which were at least 1 km in distance from each other. The locations of the sampling points are shown in Figure 2. Samples were collected from each of the sampling stations and each station consisted of three sampling points: southern, middle, and northern parts. A sample was collected from each sampling point at 20 cm depth of the water. Each sample was collected in a 1.1 L capacity volume, clean, amber glass bottle fitted with polytetrafluoroethylene (PTFE) lined screw caps. At first, the bottle was lowered slowly into the water and its cork opened by hand. When the bottle was filled with water, it was closed, drawn up carefully and marked with the desired sample label, and kept in the cool box (4 ± 1°C) prior to transfer in laboratory. Intense care was taken against contamination and loss of integrity of the sample constituents.

### 2.6. Sample Preservation and pH

After collection, the samples were preserved immediately into the laboratory according to EPA guideline described in EPA method 526 [23]. The preservatives included L-ascorbic acid (0.10 g/L) for dechlorination, ethylenediaminetetraacetic acid trisodium salt (0.35 g/L) to inhibit metal-catalyzed hydrolysis of targets, and diazolidinyl urea (1.0 g/L) as microbial inhibitor. Finally, the pH of all the samples was maintained in the range of 7.0 ± 1.0 by adding a mixture of trisbuffer salt of tris(hydroxymethyl)aminomethane (0.47 g/L) and tris(hydroxymethyl)aminomethanehydrochloride (7.28 g/L). The preserved sample bottles were stored in 4 ± 1°C temperature in the refrigerator until extraction and the extraction was done within 72 hours.

### 2.7. Sample Extraction Methodology by SPE Processor

Each 1-liter (L) sample was extracted by drawing through the 10 mL SPE cartridge containing Bond Elut C-18 (500 mg). The extraction procedure was as follows.

#### 2.7.1. Washing Cartridge

The SPE cartridge was washed with 10 mL dichloromethane (MeCl_2_) by rinsing the solvent down the sides of the cartridge and was soaked for about 1 minute and was drawn through to waste. N_2_ was passed through the cartridge at the pressure 5 psi until completely dried.

#### 2.7.2. Conditioning Cartridge

10 mL methanol (MeOH) was added to the cartridge and then some of it was slowly drawn through and the cartridge was allowed to soak for 1 minute in methanol. The cartridge was not allowed to go dry and then rinsed with 10 mL of D.I. water and most of it was drawn through to waste leaving a thin layer on the top of sorbent.

#### 2.7.3. Sample Addition

Each 1 liter (L) of sample water was added to the cartridge by using 10 mL pipette and the N_2_ pressure was adjusted to definite psi for 10 mL/min. After passing 1 L sample water from the bottle the cartridge was completely dried by flowing gentle stream of N_2_ at pressure 5 psi for about 15 minutes.

#### 2.7.4. Extract Elution

The dried cartridges were eluted with 6 mL solvent mixture of dichloromethane: hexane (1 : 2). The elution was done by N_2_ flow at pressures 1-2 psi for 0.5 mL/min. The elution part was repeated twice.

#### 2.7.5. Preconcentration of the Extract

The eluent was then water freed by adding anhydrous sodium sulfate (Na_2_SO_4_) and preconcentrated into exactly 1 mL in a graduated GC vial fitted with polytetrafluoroethylene (PTFE) lined screw caps by blowing down with a gentle stream of nitrogen gas and stored at 0°C before being analyzed by GC-MS.

### 2.8. Recovery of SPE Extraction

The recovery of the extraction procedure was carried out by replicate recovery studies. Threereplicate recovery samples were prepared for each PAH by spiking of appropriate amount of the PAH standard into the 10 mL of D.I. to reach concentrations of 5 *μ*g/mL. The samples were allowed to stand for 30 min. prior to extraction by SPE processor. Finally, the determination of percentage recovery was carried out by GC-MS system.

### 2.9. Specificity of SPE Extraction

The specificity of the extraction procedure was investigated by extraction of 1 L D.I. water followed by the full extraction methodology by SPE processor but without any PAHs and transferred into graduated amber GC vial prior to injection in GC-MS system.

### 2.10. Statistical Analysis

All statistical data, chart, and plot were obtained by Minitab 16 software.

## 3. Results and Discussion

A previously reported, developed, and validated (Table 1) GC-MS method was used to determine target PAHs from the river Buriganga. In this study, the standard curves for PAHs were generated by over the calibration ranges tested, that is, 0.50–6.50 *μ*g/mL, and gave high degree of correlation (0.987–0.999) between peak areas and concentrations of the analyte, displayed in Table 2. The standards retention time (*R*_*t*_) was 10.49, 15.83, 18.13, 18.26, and 21.51 min. in the column with respect to naphthalene, fluorene, phenanthrene, anthracene, and pyrene.

The river water samples were extracted by proposed reversed phase solid-phase extraction (RP-SPE) method and the overall results of percent recoveries (mean ± %RSD) of spiked PAHs ranging from 81.47 to 98.60% are indicating good recovery of the SPE extraction procedure, as shown in Table 3, and the procedure also found specific to the target analyte since none of the peaks appeared at the retention time of PAHs. The total extraction time was approximately 1.6 hours for 1 L water sample.

**Figure 3:**
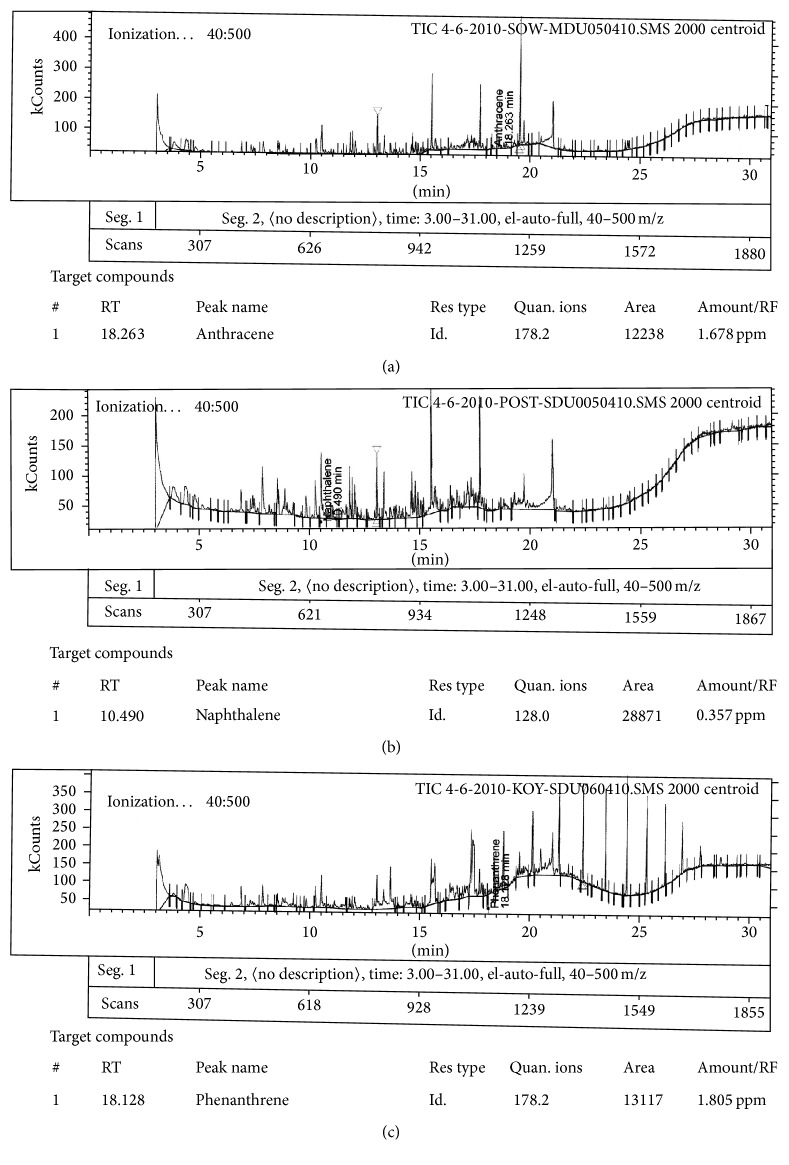
Chromatogram of the PAHs from extracted water samples: (a) anthracene, (b) naphthalene, and (c) phenanthrene.

Identification and estimation of PAHs were conducted by comparison of their retention time and mass spectra of the peaks with those standards. A series of GC-MS chromatograms of PAHs were obtained and their area was calculated from the standard calibration curve method. The individual concentrations of PAHs detected in river water collected from the different sampling locations are summarized in Table 4. The concentration levels of PAHs detected were varied from the year 2010 to 2011 at different sampling stations. The variation may be attributed to source of PAHs pollution and environmental condition. Three out of the targeted five PAHs were detected in river water in this monitoring work. The commonly found PAHs in this river water are comprised of 2-3 fused benzene rings, and they were ANT, NAP, and PHE, which indicates that these organic pollutants were mostly from anthropogenic sources within the surrounding area. The peaks of ANT, NAP, and PHE were identified in the extracted water samples by comparison of their retention times with reference standards, shown in Figure 3.

Among the three detected PAHs from the 12 sample points, the mean concentration of ANT was found utmost, 2.348–2.377 *μ*g/mL, but the total concentration level of ANT in 2011 was 16.434 *μ*g/mL, which was 4-fold higher than 2010. The temperature in Bangladesh during July (warm summer) was near 40°C, whereas in March (mild winter) the temperature was below 25°C. For instance, ANT is less soluble than NAP and PHE but the solubility of PAHs in water is enhanced three- to fourfold by a rise in temperature from 5 to 30°C. At the same time, the lowest concentration of PAH in the Buriganga river water samples was related to NAP. Maybe, vapor pressure characteristics determine the less abundance of NAP in the aquatic environment. The vapor pressure of NAP at 25°C (1.8 × 10^−2^ mm Hg) is most among all the other PAHs like ANT (2.4 × 10^−4^ mm Hg) and PHE (6.8 × 10^−4^ mm Hg). The associated mean values for NAP were in the range of 0.397–0.857 *μ*g/mL through the time and locations and the sum of detected concentration level was double in 2011 compared to 2010, maybe due to more deposition of NAP. PEH was detected as the second most concentrated PAH after ANT in water samples of the river Buriganga. The total concentration of PEH was found moderately equal between 2010 and 2011 and the mean concentration with respect to PEH was 1.219–1.248 *μ*g/mL. PHE is usually noticeable in river water; DeLeon et al. analyzed surface water from 11 locations in the Mississippi River, USA, where seventeen PAHs were identified in the samples at levels ranging from 1 ng/L for 6 compounds to 34 ng/L for phenanthrene [24]. The highest concentration of phenanthrene was detected in a sample collected near New Orleans, Louisiana, USA, near an industrial area, implicating industrial effluent or surface runoff from this area as a possible source.

**Figure 4:**
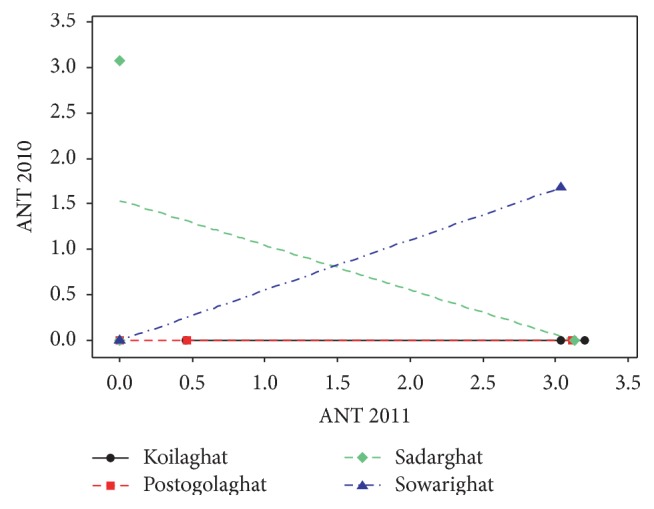
Scatter plots for anthracene (ANT) contamination in different locations (2010 versus 2011).

**Figure 5:**
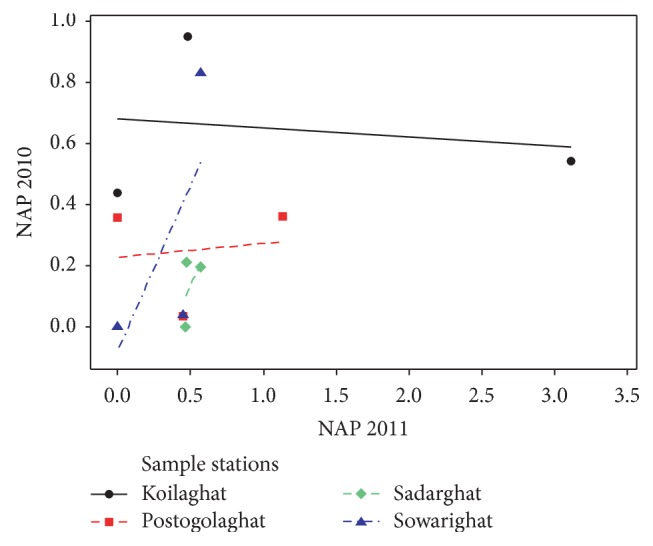
Scatter plots for naphthalene (NAP) contamination in different locations (2010 versus 2011).

**Figure 6:**
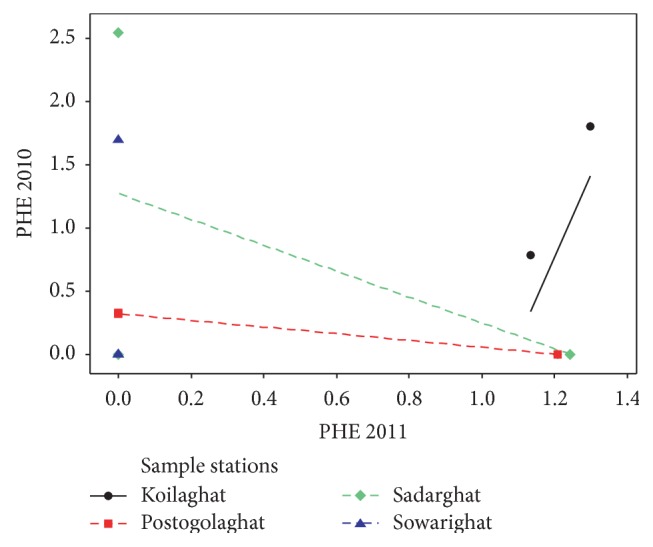
Scatter plots for phenanthrene (PHE) contamination in different locations (2010 versus 2011).

Although the PAHs were found in almost all the sampling locations, their amount varies from location to location. The middle stream of the river was assumed as the most contaminated zone with PAHs compared with the northern and southern parts. The total concentration level of ANT, NAP, and PHE in the middle stream of the river was observed to be 1.678, 1.950, and 2.873 *μ*g/mL and 3.949, 5.291, and 1.210 *μ*g/mL in 2010 and in 2011, respectively. ANT was detected in the concentration level of 3.201 *μ*g/mL at southern stream of Koilaghat station and 3.076 *μ*g/mL at southern stream of Sadarghat station as maximum in 2011 and in 2010, respectively. In a comparison of 2010 and 2011, represented in Figure 4, it was observed that the ANT contamination was detected in seven sampling points in 2011 but in 2010 ANT detection points were only two, one at middle stream of Sowarighat and the other one at southern stream of Sadarghat station. Concentration of NAP was detected to be 3.113 *μ*g/mL as maximum in 2011 at the middle stream of Koilaghat station and as minimum concentration of 0.451 *μ*g/mL at northern stream of Postogolaghat station, shown in Figure 5. Alternatively in 2010, the lowest concentration of NAP was obtained to be 0.033 *μ*g/mL at northern stream of Postogolaghat station and the highest detected level was found to be 0.952 *μ*g/mL at northern stream of Koilaghat station.  Figure 6 also displayed that the contamination level of NAP was low but it was not localized but rather spreading all over in the river. In 2011, maximum PEH concentration was obtained lower than 2010 and that was 1.298 *μ*g/mL, at southern stream of Koilaghat station. The maximum PEH contamination level was observed to be 2.546 *μ*g/mL at middle stream of Sadarghat station in 2010, as shown in Figure 6.

In this study among the five targeted PAHs, fluorene (3 rings) and pyrene (4 rings) were not detected at any location. The absence of FLU and PYE was probably due to their presence in very low amounts. Alternatively, these PAHs may be degraded or biodegraded to other constituents [25]. A broad range of microorganisms including fungi, algae, and bacteria are known to degrade PAHs. Photooxidation and rapid photolysis may be also a reason of depletion of high molecular (>3 rings) PAH like pyrene. In 1993, Hall et al. [26] analyzed 48 h composite samples from three locations in the Potomac River and three locations in the upper Chesapeake Bay for eight PAHs: perylene, fluorene, phenanthrene, anthracene, fluoranthene, pyrene, benzo[a]anthracene, and chrysene; pyrene (PYR) was detected in only one of nine Chesapeake Bay samples and not found in any of the Potomac River samples. The acute toxicity of PAHs in water appears to be a function of its diaromatic hydrocarbon (two rings), such as NAP content. In 1983, the International Joint Commission (IJC) established a limit for benzo[a]pyrene (BaP) in water to be less than 0.01 *μ*g/L and also noted that 3 to 5 PAHs are carcinogenic and may be equal.

Anthropogenic sources of PAHs in the river Buriganga are mainly pyrogenic (derived from fuel combustion) and petrogenic (derived from petroleum or crude oil contamination). The abundance of ANT, NAP, and PEH was generally accredited to shipping activities, urban runoff, oil spillages, coal burn, atmospheric deposition, untreated industrial and domestic waste discharges and effluent discharge outlets from the city (Dhaka), and biosynthesis by microorganisms like bacteria, fungi, and algae. The northern part of the river consists of launch terminal (Sadarghat and Sowarighat stations) where the launches, tankers, and boats stop for shipment, loading-unloading for goods-passengers, and often jet-black engine oil. A trade place of burnt oil is situated at southern part near the area between Sadarghat and Koilaghat stations, where oil has been sold to the oil-trader community from the launch operators, and this burnt oil is brought back to them after refining by the oil-traders. In addition, engine boats also release burnt oil into the river which is also a source of PAHs pollution. As a result, maximum petroleum products may float on the surface of the water. Including the oil trade community, the southern part (Postogolaghat to Koilaghat stations) of the rivers mainly associated with ship yards where ship repairing, maintaining, greasing, oiling, and inland navigation are periodically treated with coal tar to prevent corrosive damage. Coal tars known as manufactured gas plant residue (MGP) are complex mixtures containing over 1000 compounds, of which at least 30 are PAHs. The leftover grease and spoils are conveniently thrown into the river. Brick kilns are also a possible source of PAHs in Buriganga; there are about 300 brick kilns draining their wastes into this river.

## 4. Conclusions

The proposed reversed phase solid-phase extraction (RP-SPE) procedure for PAHs extraction from water samples is cost-effective, simple, precise, and reproducible. In this study, three low-molecular-weight PAHs out of five targeted PAH compounds were found, which reflect that acute anthropogenic sources are the possible origin of these organic pollutants that discharged into the rivers. The US EPA did not establish PAH criteria for the protection of aquatic life, except for BaP in water, which should be less than 0.01 *μ*g/L. According to guidelines of Environment Canada [27], the interim levels of ANT, NAP, and PHE for the protection of aquatic life are 0.012, 1.1, and 0.4 *μ*g/L, respectively. Results obtained with the present study provided contamination level of low-molecular-weight (LMW) PAH in Buriganga river which is significantly higher than the guidelines value.

## Supplementary Material

The targeted reversed phase SPE extracted PAHs from the river Buriganga were determined by a validated GC-MS method. The linearity results of the calibration curves showed that an excellent correlation exists between peak areas and concentrations of the PAHs. The detected PAH compounds from the different locations of the river water were anthracene, naphthalene, and phenanthrene at the concentration ranges of 0.451 to 3.201, 0.033 to 3.1131, and 0.320 to 2.546 μg/mL, respectively. The peaks of anthracene, phenanthrene, and naphthalene were confirmed by comparison of their retention times with reference standards.

## Figures and Tables

**Table 1:** Method validation summary of the GC-MS method [22].

PAHs	Retention time (min.)	Linear regression coefficient (*R* ^2^)	Detection limit ng/mL	Precision (%) (SD^2^) (*n* = 5, 10 *μ*g/mL)	
				Interday	Intraday
ANT	18.27	0.996	2.50	1.43	0.67
FLU	15.87	0.999	2.30	0.78	0.42
NAP	10.53	0.998	2.00	0.63	0.34
PHE	18.17	0.987	2.00	0.81	0.29
PYR	21.55	0.998	3.10	2.34	0.74

**Table 2:** Linearity and calibration curve data of PAHs standard solution by GC-MS.

PAHs	Retention time (min.)	Ion monitored (*m*/*z*)	Year 2010		Year 2011	
			Linear equation	*R* ^2^	Linear equation	*R* ^2^
ANT	18.26	178	*y* = 6982.45*x* − 643.49	0.994	*y* = 7155.70*x* − 1868.08	0.987
FLU	15.83	165	*y* = 9015.00*x* − 100.00	0.989	*y* = 9239.45*x* − 799.30	0.996
NAP	10.49	128	*y* = 3383.70*x* − 1844.00	0.999	*y* = 3164.50*x* − 1170.43	0.999
PHE	18.13	178	*y* = 6904.82*x* − 652.42	0.993	*y* = 6878.68*x* − 580.16	0.989
PYR	21.51	202	*y* = 1787.18*x* − 208.09	0.987	*y* = 1727.208*x* − 199.10	0.990

**Table 3:** The percentage recoveries of PAHs extraction by C-18 solid-phase with solvent mixture of dichloromethane : hexane (1 : 2).

PAHs	Amount added (*μ*g/mL)	Retention time	Area	Amount recovered (*μ*g/mL)	% Recovery	% Recovery (mean ± %RSD)
ANT	5.00	18.13	30294	4.58	91.60	89.73 ± 1.82
	5.00	18.13	29865	4.45	89.00	
	5.00	18.13	30867	4.43	88.60	

FLU	5.00	15.83	102408	4.90	98.00	98.60 ± 0.61
	5.00	15.82	103662	4.96	99.20	
	5.00	15.83	103035	4.93	98.60	

NAP	5.00	10.47	227059	4.02	80.40	81.47 ± 1.16
	5.00	10.47	231013	4.09	81.80	
	5.00	10.47	232143	4.11	82.20	

PHE	5.00	18.27	32503	4.23	84.60	84.73 ± 1.66
	5.00	18.27	31580	4.17	83.40	
	5.00	18.27	31438	4.31	86.20	

PYR	5.00	21.51	17145	4.77	95.40	96.07 ± 1.03
	5.00	21.51	17469	4.86	97.20	
	5.00	21.51	17181	4.78	95.60	

**Table 4:** Concentration (*µ*g/mL) of PAHs in 20 cm depth water samples collected from Buriganga river in 2010 and in 2011.

Sample stations	Sample points	Year 2010			Year 2011		
		PAHs (*µ*g/mL)					
		ANT	NAP	PHE	ANT	NAP	PHE
Koilaghat	Northern	×	0.952	0.789	3.036	0.484	1.133
	Middle	×	0.544	×	0.451	3.113	1.210
	Southern	×	0.440	1.805	3.201	×	1.298

Sowarighat	Northern	×	×	1.702	×	×	×
	Middle	1.678	0.831	×	3.036	0.572	×
	Southern	×	0.038	×	×	0.451	×

Sadarghat	Northern	×	0.197	×	3.135	0.572	1.243
	Middle	×	0.212	2.546	×	0.473	×
	Southern	3.076	×	×	×	0.462	×

Postogolaghat	Northern	×	0.033	×	3.113	0.451	1.210
	Middle	×	0.363	0.327	0.462	1.133	×
	Southern	×	0.357	0.320	×	×	×

The sign “×” stands for not detected.
