# Supplementary material for: Reversed Phase SPE and GC-MS Study of Polycyclic Aromatic Hydrocarbons in Water Samples from the River Buriganga, Bangladesh
Source: Int Sch Res Notices. 2014 Oct 29;2014:234092. doi: 10.1155/2014/234092 (PMC4908253; doi:10.1155/2014/234092)
Supplement: Supplementary file 1 — The targeted reversed phase SPE extracted PAHs from the river Buriganga were determined by a validated GC-MS method. The linearity results of the calibration curves showed that an excellent correlation exists between peak areas and concentrations of the PAHs. The detected PAH compounds from the different locations of the river water were anthracene, naphthalene, and phenanthrene at the concentration ranges of 0.451 to 3.201, 0.033 to 3.1131, and 0.320 to 2.546 μg/mL, respectively. The peaks of anthracene, phenanthrene, and naphthalene were confirmed by comparison of their retention times with reference standards. [file 234092.f1.pdf]

SUPPLEMENTARY MATERIALS

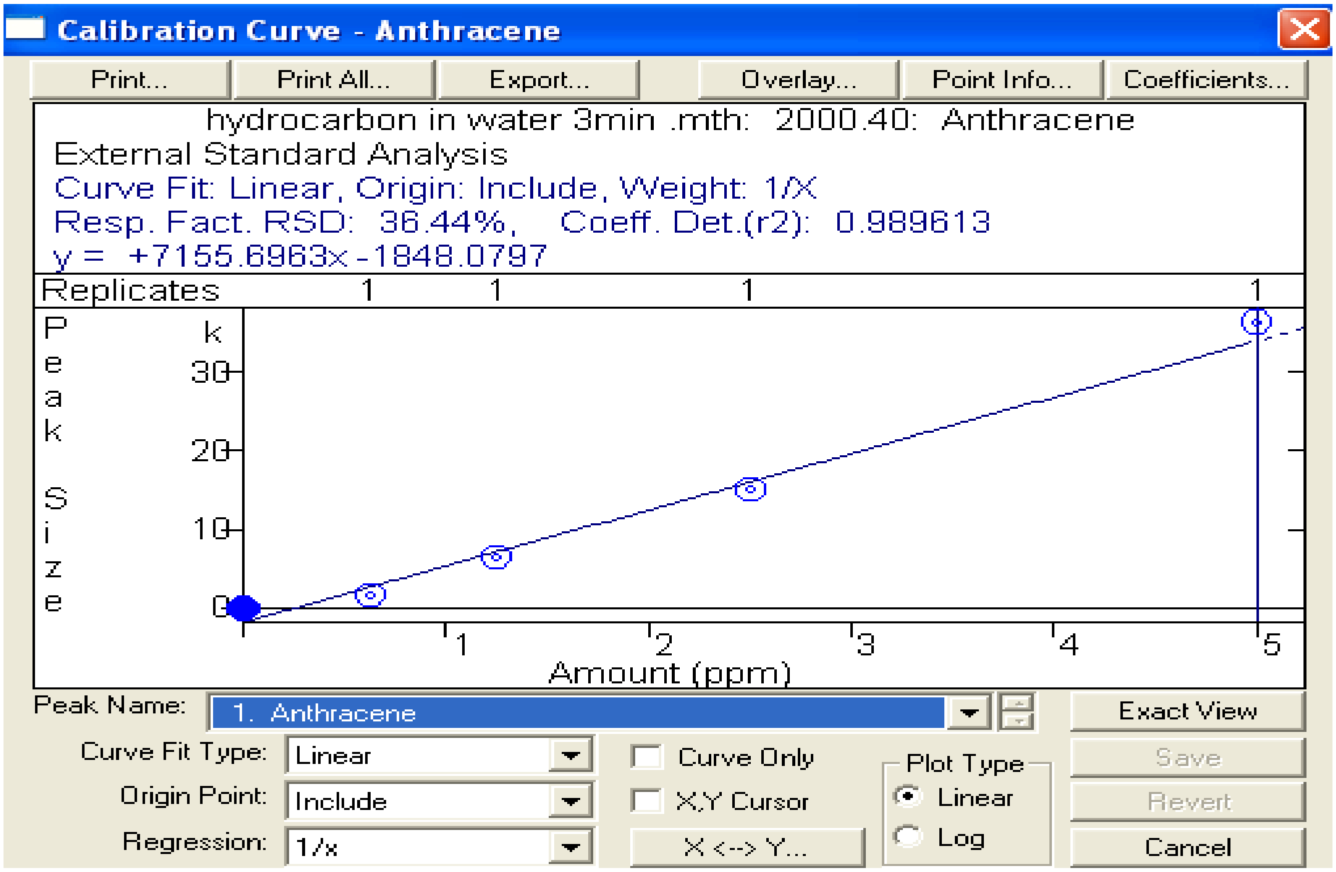

Fig: Calibration curve Anthracene (ANT), year 2011

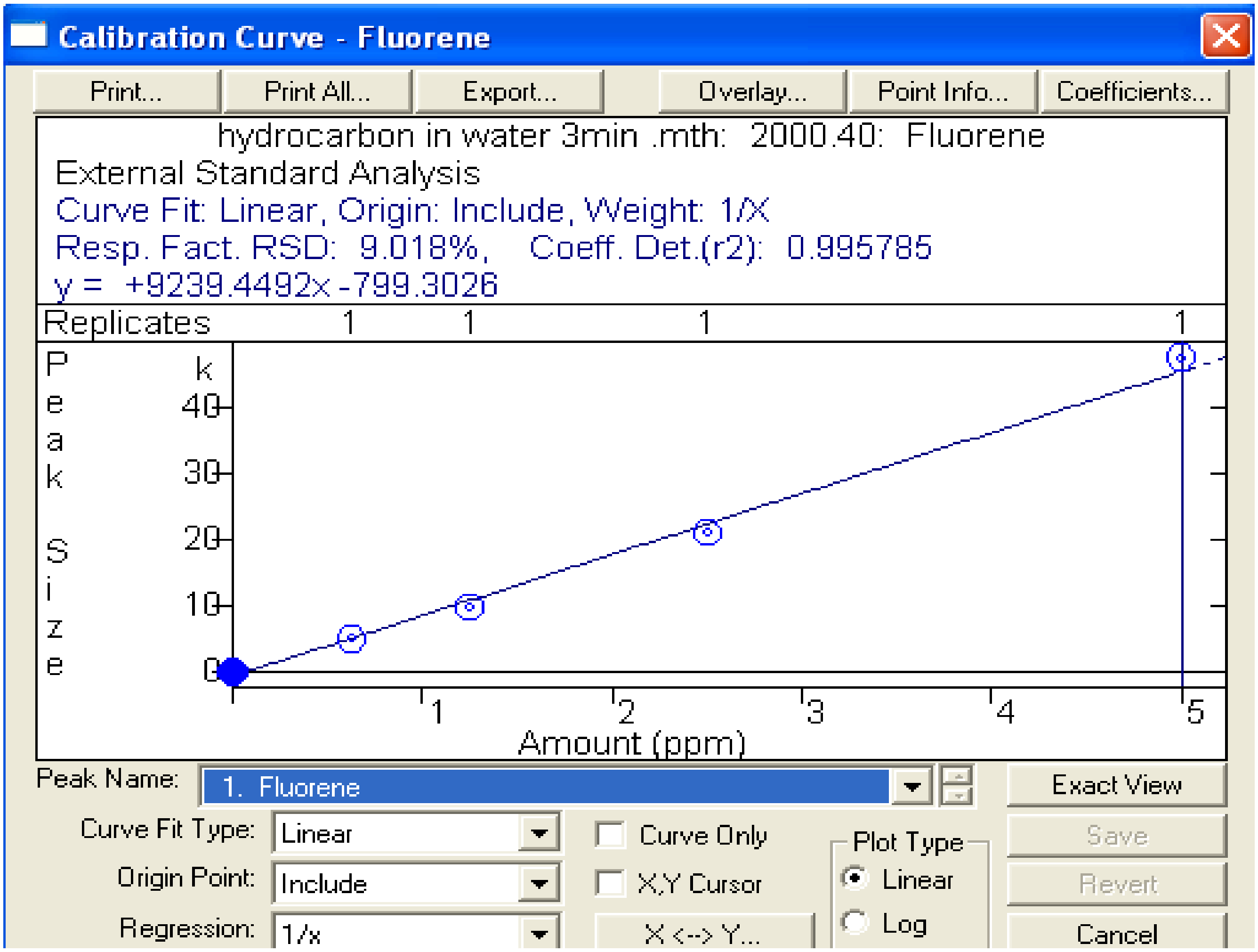

Fig: Calibration curve Fluorene (FLU), year 2011

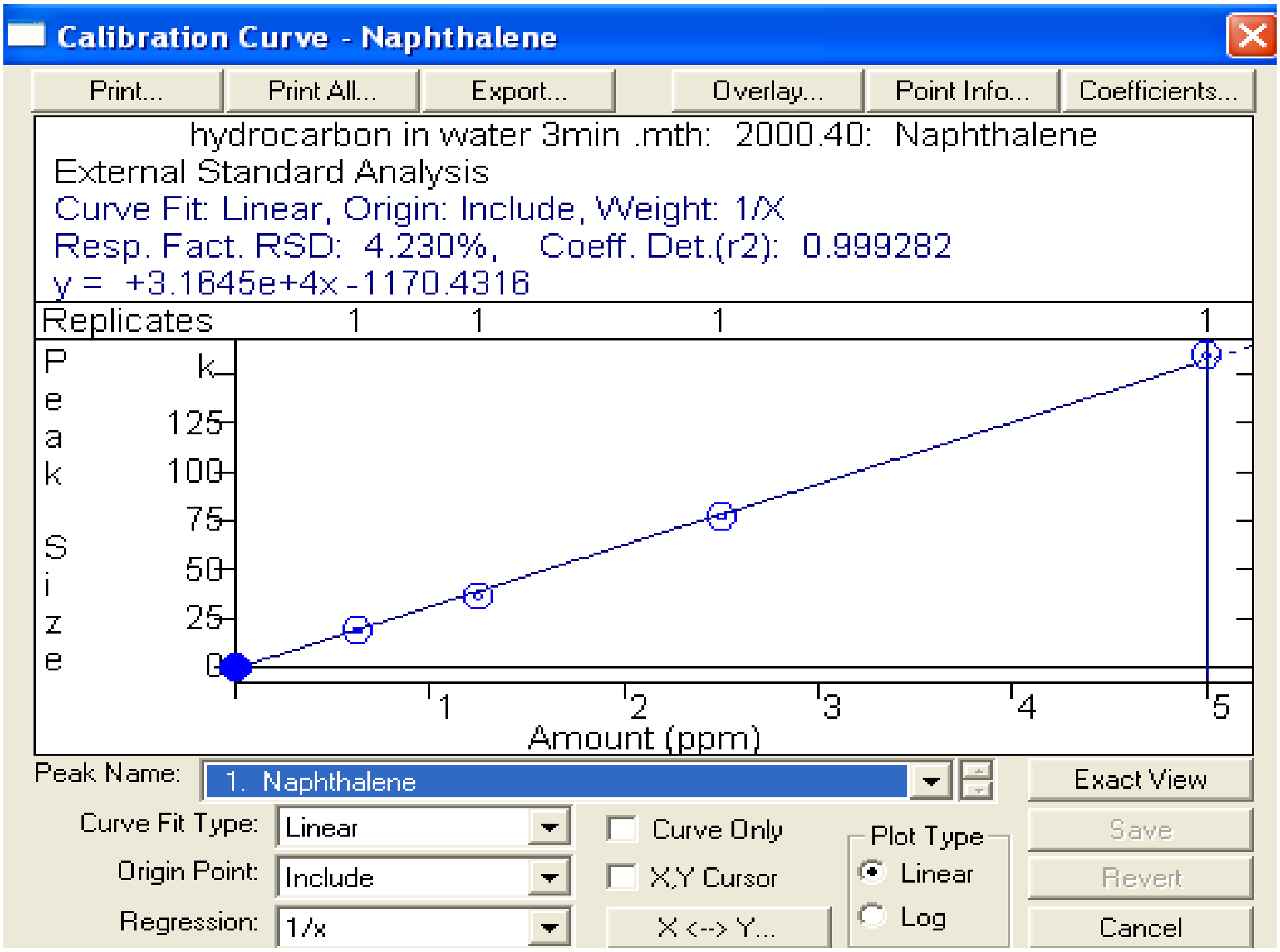

Fig: Calibration curve Naphthalene (NAP), year 2011

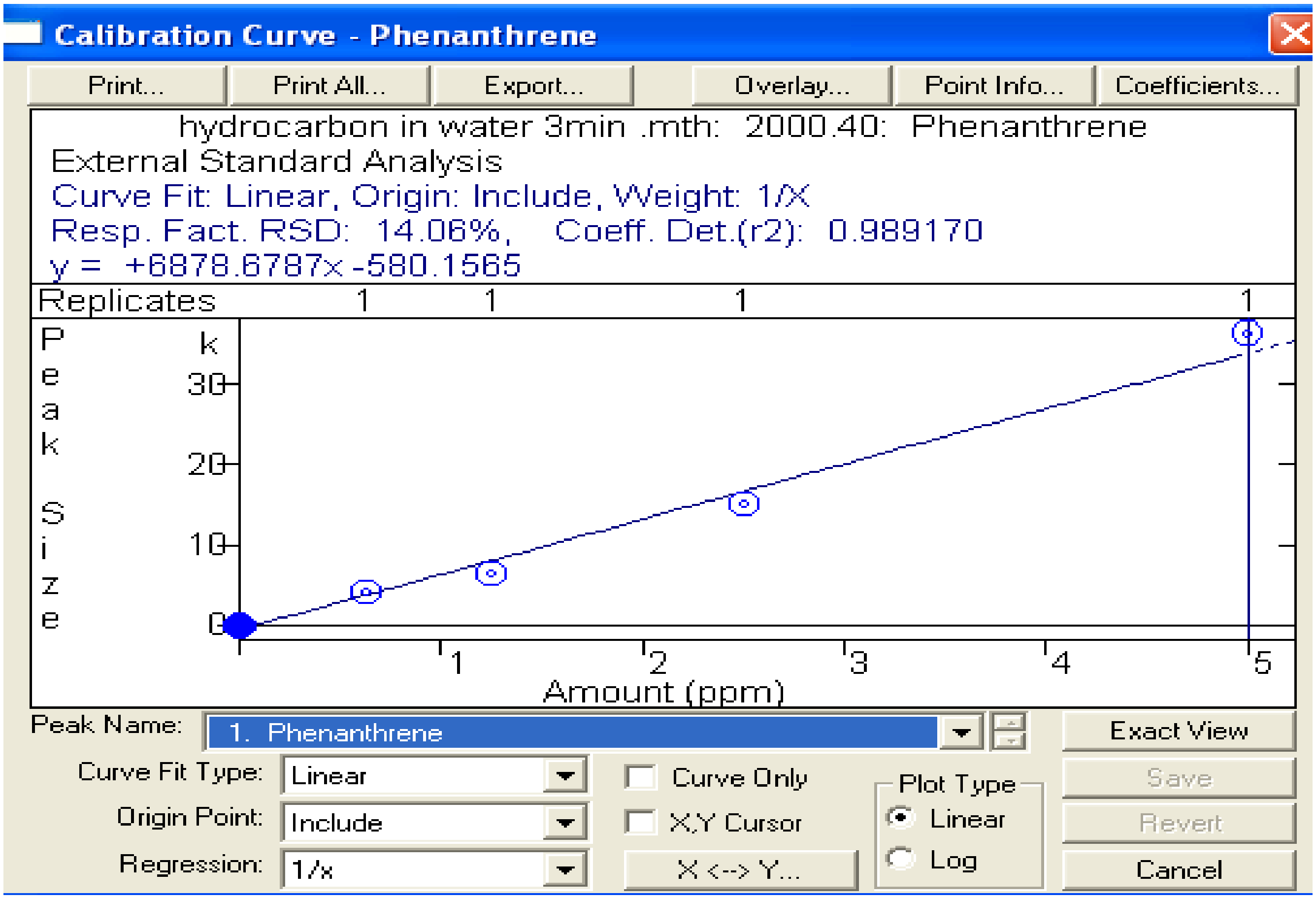

Fig: Calibration curve Phenanthrene (PHE), year 2011

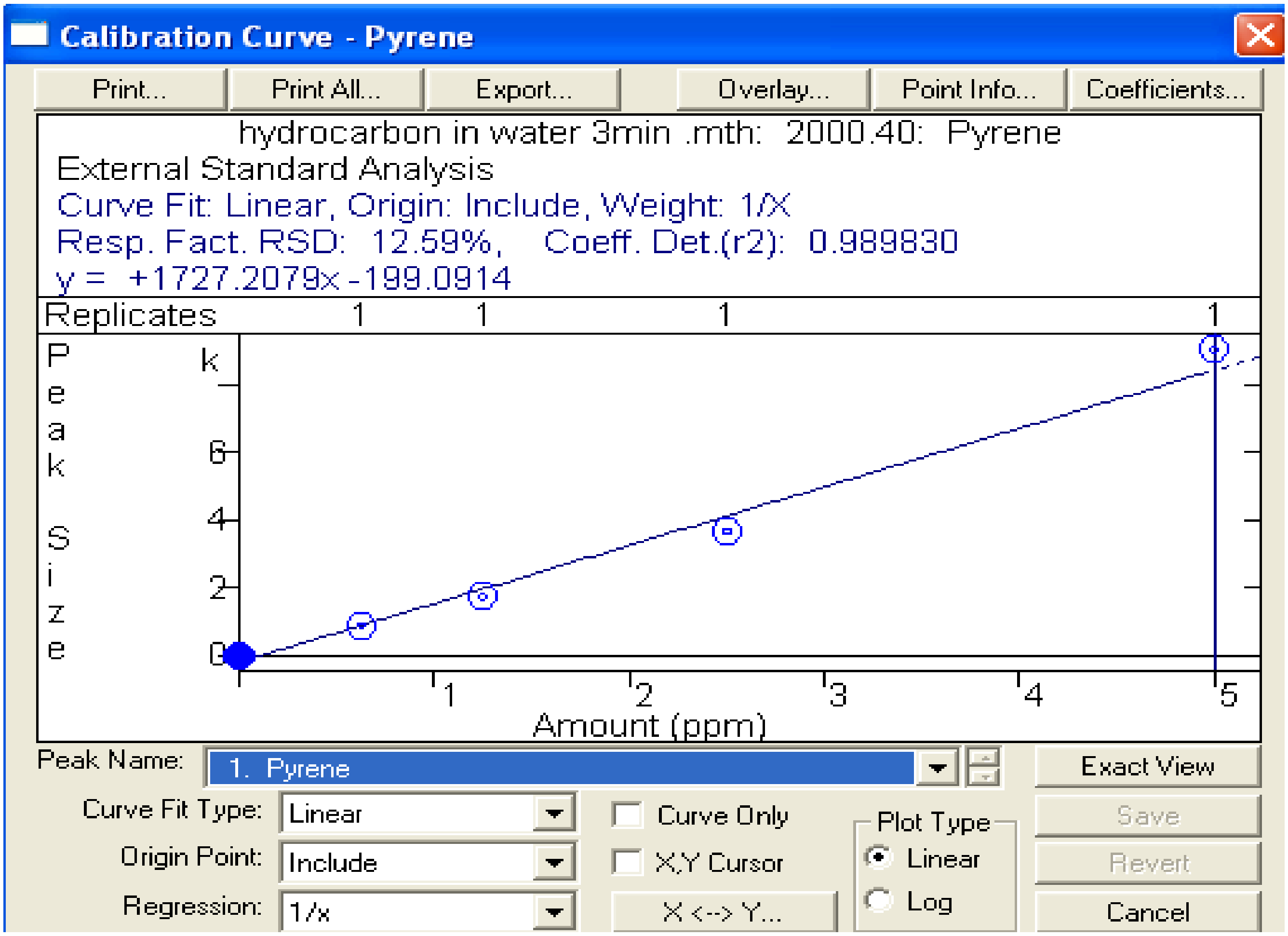

Fig: Calibration curve Pyrene (PYE), year 2011

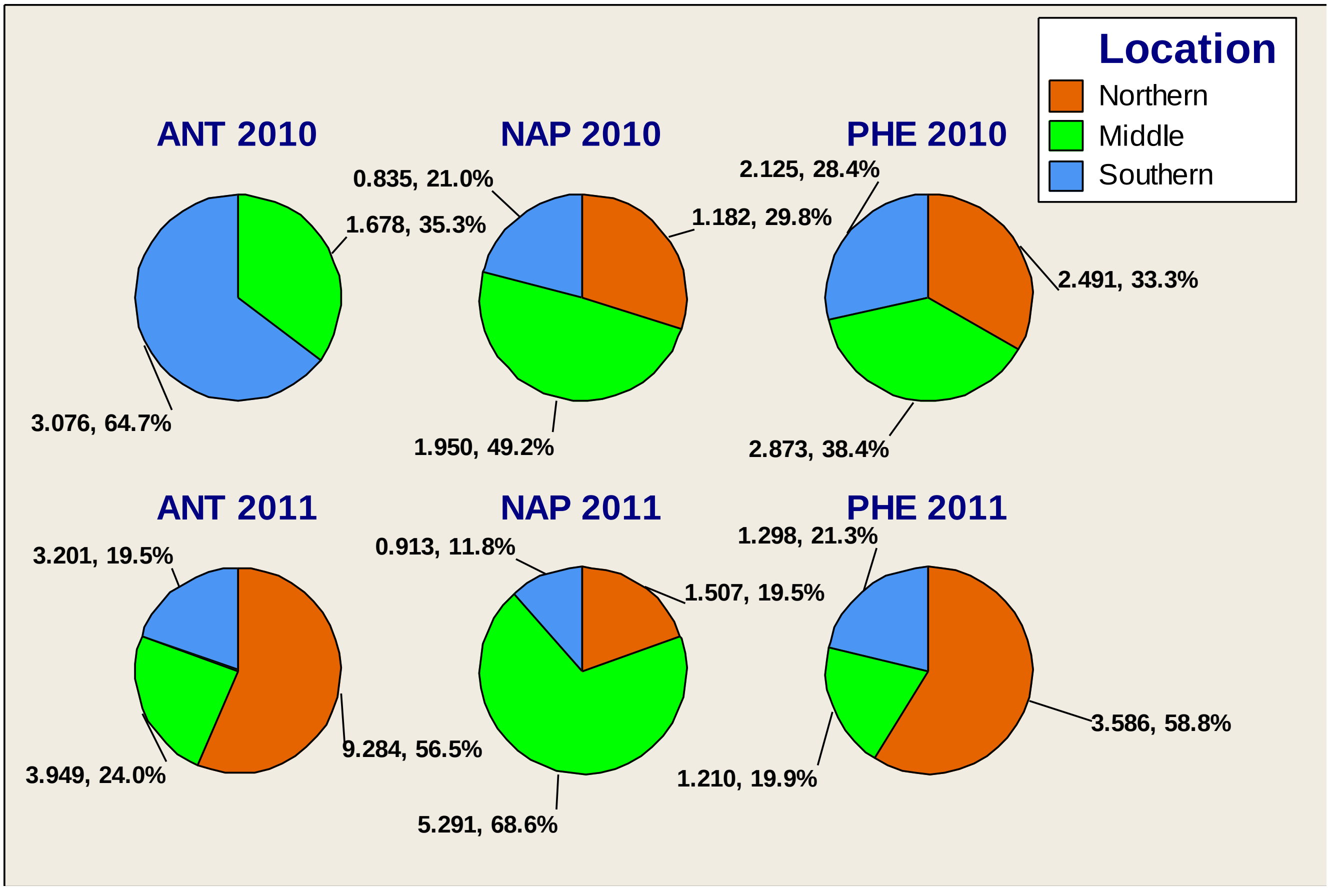

Fig. : Concentration (µg/mL, %) of individual PAHs in 2010 and in 2011 at northern, middle and southern sides of the river Buriganga.
